# Enhancing Intrusion Detection Systems for IoT and Cloud Environments Using a Growth Optimizer Algorithm and Conventional Neural Networks

**DOI:** 10.3390/s23094430

**Published:** 2023-04-30

**Authors:** Abdulaziz Fatani, Abdelghani Dahou, Mohamed Abd Elaziz, Mohammed A. A. Al-qaness, Songfeng Lu, Saad Ali Alfadhli, Shayem Saleh Alresheedi

**Affiliations:** 1 School of Computer Science and Technology, Huazhong University of Science and Technology, Wuhan 430074, China; 2 Computer Science Department, Umm Al-Qura University, Makkah 24381, Saudi Arabia; 3 Faculty of Computer Sciences and Mathematics, Ahmed Draia University, Adrar 01000, Algeria; 4 Department of Mathematics, Faculty of Science, Zagazig University, Zagazig 44519, Egypt; 5 Artificial Intelligence Research Center (AIRC), Ajman University, Ajman 346, United Arab Emirates; 6 Department of Artificial Intelligence Science and Engineering, Galala University, Suze 435611, Egypt; 7 Department of Electrical and Computer Engineering, Lebanese American University, Byblos 13-5053, Lebanon; 8 College of Physics and Electronic Information Engineering, Zhejiang Normal University, Jinhua 321004, China; 9 Hubei Engineering Research Center on Big Data Security, School of Cyber Science and Engineering, Huazhong University of Science and Technology, Wuhan 430074, China; 10 Shenzhen Huazhong University of Science and Technology Research Institute, Shenzhen 518057, China; 11 Department of Computer Techniques Engineering, Imam Al-Kadhum College, Baghdad 10081, Iraq; 12 War College, National Defense University, Riyadh 12211, Saudi Arabia

**Keywords:** metaheuristics, cyber security, intrusion detection system, Internet of Things (IoT), Growth Optimizer, CNNs

## Abstract

Intrusion detection systems (IDS) play a crucial role in securing networks and identifying malicious activity. This is a critical problem in cyber security. In recent years, metaheuristic optimization algorithms and deep learning techniques have been applied to IDS to improve their accuracy and efficiency. Generally, optimization algorithms can be used to boost the performance of IDS models. Deep learning methods, such as convolutional neural networks, have also been used to improve the ability of IDS to detect and classify intrusions. In this paper, we propose a new IDS model based on the combination of deep learning and optimization methods. First, a feature extraction method based on CNNs is developed. Then, a new feature selection method is used based on a modified version of Growth Optimizer (GO), called MGO. We use the Whale Optimization Algorithm (WOA) to boost the search process of the GO. Extensive evaluation and comparisons have been conducted to assess the quality of the suggested method using public datasets of cloud and Internet of Things (IoT) environments. The applied techniques have shown promising results in identifying previously unknown attacks with high accuracy rates. The MGO performed better than several previous methods in all experimental comparisons.

## 1. Introduction

The need to secure online data, information, and related systems has grown in importance with the development of information communication, particularly the Internet. Sensitive data is created, transported, stored, or updated worldwide daily in enormous quantities. Private emails, financial transactions, simple holiday photos, and military communications are all examples of sensitive information. Malicious parties have sought to steal, alter, or erase this information for a long time. Hackers and other hostile actors have developed, exploited, and enhanced various cyberattacks to accomplish these objectives [1]. A paradigm shift from straightforward defense mechanisms to complex defense systems was necessary for this new era of cyber security. While simple network security measures such as firewalls may have been enough in the past, the sophistication of cyberattacks has made them ineffective when used alone. Intrusion Detection Systems (IDS) are currently the cornerstone of cyber security to defend against these sophisticated attacks [1]. In the cloud and IoT, there are three primary divisions of cloud services: infrastructure-as-a-service (IaaS), platform-as-a-service (PaaS), and software-as-a-service (SaaS). To give users security, it is necessary to address the weaknesses and problems each of these services and methods possesses [2]. In recent years, different methods have been proposed for the IDS, such as the traditional machine learning techniques, for example, the support vector machine (SVM) [3,4], decision trees [5,6], k-means clustering [7,8], and others. The recent advances in deep neural networks, including conventional neural networks (CNNs) and recurrent neural networks (RNNs), were also adopted in this field [9]. Several IDS were developed based on ANNs, such as RNNs [10] and CNNs [11].

In recent years, a new direction was utilized for the IDS by employing the power of the metaheuristic optimization algorithms adopted in different and complex engineering and optimization problems, including IDS. For example, Alazab et al. [12] employed the moth–flame optimizer algorithm to build an IDS method. The MFO was a feature selection method that enhanced the classifier’s performance (Decision Tree). The evaluation showed that the classification accuracy of the DT was improved by applying the MFO. In [13], the authors applied a combined MH method using the firefly algorithm (FA) and ant lion optimization algorithm to build an efficient IDS system. Zhou et al. [14] employed the bat algorithm as a feature selection to build an IDS. It was evaluated with random forest classifier, C4.5, and ForestPA. It is clear that MH optimization algorithms have shown significant performance in IDS applications; thus, they have been widely adopted, such as whale optimization algorithm [15], particle swarm optimization algorithm [16], Aquila optimization algorithm [17], reptile search algorithm [18], salp swarm algorithm [19], and many others.

### Paper Contribution

Following the successful applications of MH optimization algorithms in IDS, we propose an efficient feature selection technique called MGO. This method is developed based on two aspects; the first is to utilize the power of the Growth Optimizer (GO) in the exploration phase of the search process. The second aspect is to employ the integration between GO and WOA in the exploitation phase. The main objective of this study can be simplified as the following points:

1. Suggest a different method for securing IoT by combining DL and feature selection techniques.
2. Use a CNN model to analyze network traffic records and identify complex feature representations.
3. Create a modified version of Growth Optimizer (GO) for improved intrusion detection in IoT environments. The modification uses the operators of the Whale Optimization Algorithm (WOA). The proposed method, called MGO, is employed to address the issue of discrete feature selection.
4. Evaluate the performance of the MGO against established methods using four actual intrusion datasets.

The paper is structured as follows: Section 2 explains the employed methods, Section 3 outlines the proposed IoT security system, Section 4 assesses the system, and Section 5 concludes the results.

## 2. Background

### 2.1. Growth Optimizer

In this section, the Growth Optimizer (GO) simulates how people train and reflect as they progress in society. In the learning phase, the information is collected from the environment, whereas the reflection aims to examine the shortcomings and improve the learning method.

In general, the GO starts by using Equation (1) to generate the population *X* which stands for the solutions for the tested problem.

(1) $$X_{i}=r\times \left(U-L\right)+L,\;i=1,...,N$$

where *r* is the random value and the limits of the search domain of the problem are represented using *U* and *L*. *N* refers to the total number of solutions in *X*.

Following [20], *X* is divided into three parts according to the parameters named $P_{1}=5$. The first part comprises the leader and the elites (varying from 2 to $P_{1}$). The second part contains the middle level (i.e., from $P_{1}+1$ to $N-P_{1}$) and the third part contains the bottom level (i.e., $N-P_{1}+1$ and *N*), whereas the best solution is the leader of the upper level.

#### 2.1.1. Learning Stage

By confronting disparities between people, examining the causes of those differences, and learning from them, individuals can be greatly helped in their progress. The GO’s learning stage simulates four key gaps that are formulated:

(2) $$\begin{array}{cc} \; & G_{1}=X_{b}-X_{bt}\\ \; & G_{2}=X_{b}-X_{w}\\ \; & G_{1}=X_{bt}-X_{w}\\ \; & G_{1}=X_{r1}-X_{r2} \end{array}$$

where $X_{b},X_{bt},X_{w}$ indicate best, better, and worst solution, respectively; in addition, $X_{r1}$, and $X_{r2}$ are two random solutions. $G_{k}\left(k=1,2,3,4\right)$ stands for the gap used to improve the skills learned and decrease the difference between them. Moreover, to reflect the variation between the groups, the parameter named learning factor (LF) is applied and its formulation is given as:

(3) $$LF_{k}=\frac{\left|\right|G_{k}\left|\right|}{\sum_{k=1}^{4}\left|\right|G_{k}\left|\right|},k=1,2,3,4$$

Following [20], the individual can assess his learned knowledge using the parameter ($SF_{i}$):

(4) $$SF_{i}=\frac{GR_{i}}{GR_{max}}$$

where $GR_{max}$ and $GR_{i}$ represent the maximum growth resistance of *X* and the growth of $X_{i}$, respectively.

According to the information collected from $LF_{k}$ and $SF_{i}$ each $X_{i}$ can receive new knowledge from the solution belonging to each gap $G_{k}$ using the knowledge acquisition ($KA_{k}$) that is defined as:

(5) $$KA_{k}=SF_{i}\times LF_{k}\times G_{k},\;k=1,2,3,4$$

After that, the solution $X_{i}$ can improve its information using the following formula:

(6) $$X_{i}\left(t+1\right)=X_{i}\left(t\right)+\sum_{k=1}^{4}KA_{k}$$

The quality of the updated version of $X_{i}$ is computed and compared with the previous one to determine whether there is a significant difference between them.

(7) $$X_{i}\left(t+1\right)=\left\{\begin{array}{cc} X_{i}\left(t+1\right) & if\;f\left(X_{i}\left(t+1\right)\right)\leq f\left(X_{i}\left(t\right)\right)\\ \left\{\begin{array}{cc} X_{i}\left(t+1\right) & if\;\;r_{1}<P_{2},ind\left(i\right)=ind\left(1\right)\\ X_{i}\left(t\right) & otherwise \end{array}\right. & otherwise \end{array}\right.,$$

where $r_{2}$ stands for a random number and $P_{2}=0.001$ is the probability retention. ind(i) refers to the ranking of $X_{i}$ based on the ascending order *X* using the fitness value.

#### 2.1.2. Reflection Stage

The solution must develop their ability to reflect on the knowledge they have learned, meaning that *X* must identify all of their areas of weakness, make up for them, and retain their information. They ought to adopt the undesirable attributes of successful *X* while retaining their outstanding qualities. When the lesson of a specific aspect cannot be mended, the prior information should be abandoned and systematic learning should resume. Equations (8) and (9) can be used to mathematically model this process.

(8) $$X_{i}\left(t+1\right)=\left\{\begin{array}{cc} \left\{\begin{array}{cc} r_{4}\times \left(U-L\right) & if\;\;r_{3}<AF\\ X_{i}\left(t\right)+r_{5}\times \left(X_{R}-X_{i}\left(t\right)\right) & otherwise \end{array}\right. & otherwise\\ X_{i}\left(t\right) & otherwise \end{array}\right.,$$

(9) $$AF=0.01+0.99\times (1-\frac{FEs}{max_{FE}})$$

where $r_{3},r_{4}$, and $r_{5}$ are random values. $X_{R}$ refers to a solution defined as the top $P_{1}+1$ solutions in *X*. AF refers to the attenuation factor which depends on function evaluation FE and the total number of functions evaluations $max_{FE}$.

After the complete reflection stage, $X_{i}$ should evaluate its growth, similar to the learning phase. Therefore, Equation (7) is also applied to achieve this task.

### 2.2. Whale Optimization Algorithm

The WOA [21] draws inspiration from the unique hunting strategy used by a particular species of killer whale known as humpback, whose hunting style is bubble-net feeding. WOA’s mathematical formulation depends on how it behaves when hunting. Each whale’s location can be represented by the solution $X_{b}$, which can be updated depending on how the whale behaves when attacking its prey. The whales can attack their prey using two different methods. The humpback whale locates its prey and encircles it using the first strategy, known as encircling prey. WOA presupposes that the target prey is the best option ($X_{b}\left(t\right)$). The other whales attempt to update their locations in the direction of $X_{b}\left(t\right)$ after it has been identified (found), as in Equation (10):

(10) $${Dis}_{i}=\left|B⊙X_{b}\left(t\right)-X_{i}\left(t\right)\right|,\;B=2r$$

(11) $$X_{i}\left(t+1\right)=X_{b}\left(t\right)-A⊙{Dis}_{i},\;A=2a⊙r-a$$

where ${Dis}_{i}$ stands for the distance between $X_{i}\left(t\right)$ and $X_{b}\left(t\right)$. r∈[0,1] refers to a random value. In addition, *a* denotes a parameter that decreases from 2 to 0 during the process of updating the solution, formulated as $a=a-t\frac{a}{t_{max}}$ ($t_{max}$ is the total of iterations).

The second strategy is called the bubble-net attack. This phase has two main steps: spiral updating location and shrinking encircling mechanism, and reducing the value of *a* in Equation (11) for satisfying the process of shrinking encircling. The whale’s locations, $X_{i}$ and $X_{b}$, are separated by the following distance, which is calculated by the spiral updating position method [21]:

(12) $$X\left(t+1\right)={Dis}^{\prime }⊙e^{bl}⊙cos\left(2\pi l\right)+X_{b}\left(t\right)$$

In Equation (12), *l* stands for a constant value which represents the shape of the logarithmic spiral.

The whales can also swim simultaneously around the $X_{b}$ utilizing a spiraling path and a contracting circle. The following equation depends on integrating Equations (10)–(11) and Equation (12) [21]; therefore, *X* can be enhanced as:

(13) $$X\left(t+1\right)=\left\{\begin{array}{cc} X_{b}\left(t\right)-A⊙Dis & if\;\;p\geq 0.5\\ {Dis}^{\prime }⊙e^{bl}⊙cos\left(2\pi l\right)+X_{b}\left(t\right) & if\;\;p<0.5 \end{array}\right..$$

In Equation (13), p∈[0,1] refers to a probability value used to identify the strategy of updating. In addition, $X_{i}$ can be enhanced using a random selecting solution $X_{r}$ instead of $X_{b}$ as represented using Equation (14) [21]:

(14) $$X\left(t+1\right)=X_{r}-A⊙Dis$$

(15) $$Dis=|B⊙X_{rand}-X(t)|$$

## 3. Proposed Method

The steps of the developed IoT security are introduced in this section. The developed technique depends on improving the performance of the Growth Optimizer using the Whale Optimization Algorithm (WOA).

### 3.1. Prepare IoT Dataset

The developed MGO starts by preparing the IoT dataset by normalizing it. This is performed using the min−max technique that applied to the IoT data DS [22], which is represented as

(16) $$\begin{array}{c} DS=\left[\begin{array}{cccc} ds_{11} & ds_{12} & ... & ds_{1d}\\ ds_{21} & ds_{22} & ... & ds_{2d}\\ ... & ... & ... & ...\\ ds_{n1} & ds_{n2} & ... & ds_{nd} \end{array}\right] \end{array}$$

where $DS_{i}=\left[ds_{i1},ds_{i2},...,ds_{id}\right]$ denotes the features of traffic *i*, whereas *n* and *d* are sample and feature numbers, respectively. The normalized version of DS based on min−max technique is represented as [22]:

(17) $$\begin{array}{c} NDS_{ij}=\frac{Ds_{ij}-min\left(DS_{j}\right)}{max\left(DS_{j}\right)-min\left(DS_{j}\right)} \end{array}$$

### 3.2. CNN for Feature Extraction

Convolutional neural networks (CNNs) are widely used in computer vision as they are robust feature extraction modules, especially when employing pre-trained models alongside transfer learning methods. Meanwhile, CNN is also used in applications where the data are one-dimensional such as in natural language processing. Our study aims to train a DL model that benefits from the big data generated from IoT devices to perform network intrusion detection and reduce processing complexity and inference time. Thus, this section proposes a light CNN model to automatically learn helpful patterns/representations rather than relying on the raw data collected from experimental and real network intrusion detection experiments. In addition, we extract the learned features for further processing (feature selection) to improve the overall framework performance (detection accuracy) and reduce the dimensionality space of the represented feature to accelerate the inference process.

**Figure 1:**
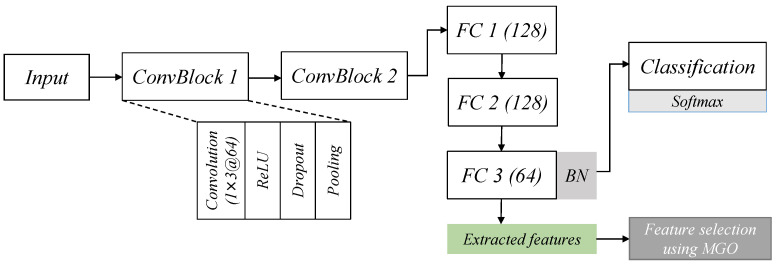
The CNN architecture employed for feature extraction.

The proposed CNN architecture receives a set of samples, *X*, where each row is a one-dimensional raw sample representing a network traffic record which includes several network attributes (columns) related to the possible attack class, such as flags related to the IP address, TCP flags, destination, source information, type of service, communication protocols, and protection protocols. The CNN architecture, as shown in Figure 1, is composed of two convolution blocks (ConvBlock) to learn spatial relations between raw attributes and generate new representations as output (feature maps). Each ConvBlock comprises a convolutional layer with a one-dimensional kernel *k*, activation function, and pooling operation. Each ConvBlock uses a kernel of size 1×3 and 64 output channels to produce the output feature maps out(t) which is a new transformation of the input raw data at a certain timestamp *t* where *i* is the input channel. A non-linear activation function name rectified linear unit (ReLU) after each convolution operation is followed by a max-pooling with size two to output the final feature maps.

### 3.3. Feature Selection-Based MGO Approach

**Figure 2:**
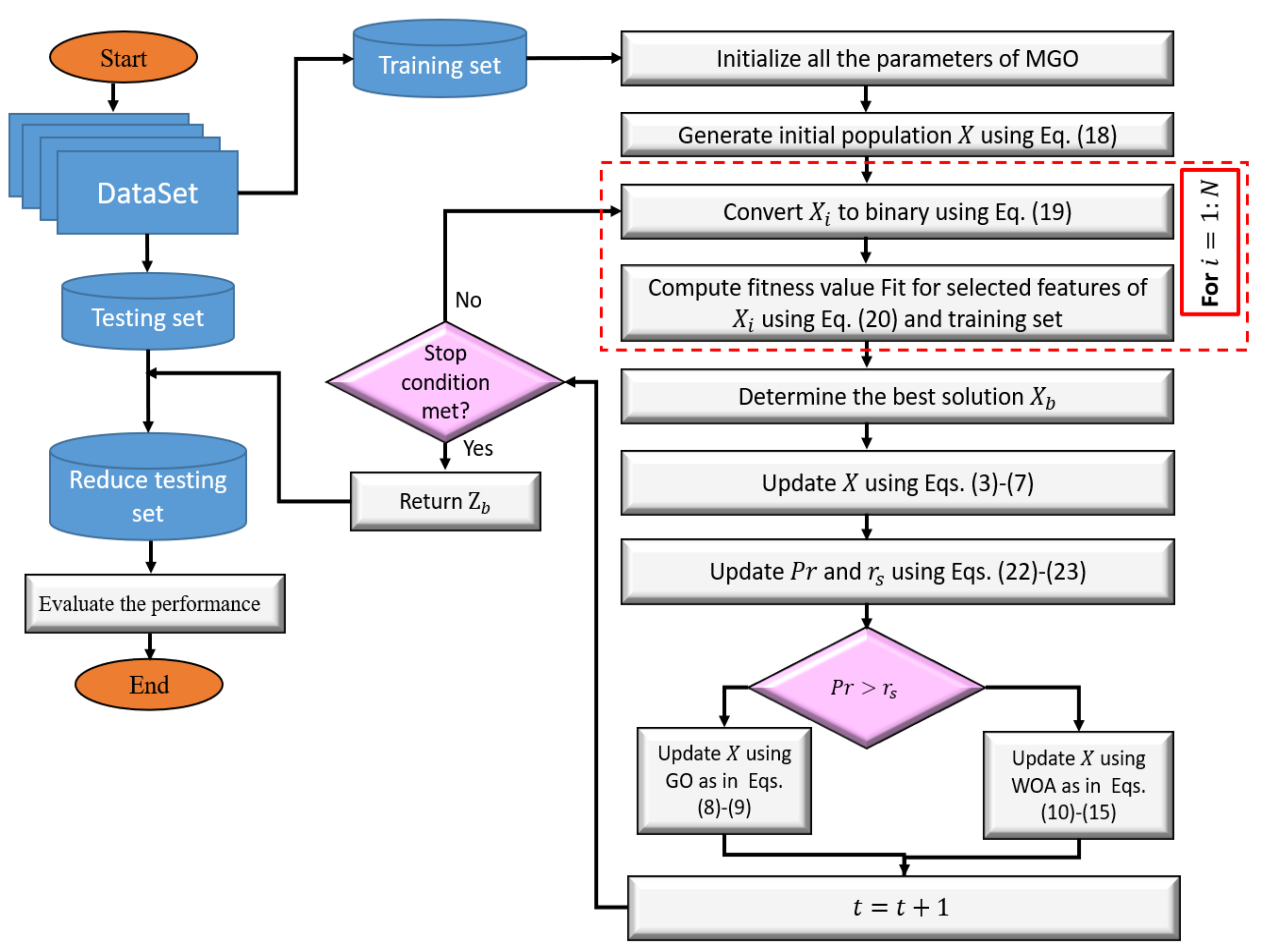
The workflow structure of the MGO feature selection technique.

We developed an alternative FS approach based on a modified version of GO algorithm using WOA as given in Figure 2. This algorithm allocates the relevant features from those extracted using the CNN model.

The first step in MGO as FS approach is to split the data into training and testing sets, which represent 80% and 20%, respectively. Then, the initial solution *X* is built as given in Equation (18).

(18) $$X_{i}=LB+rand\left(1,D\right)\times \left(UB-LB\right),\;i=1,2,...,N$$

where *N* stands for the total number of solutions and *D* the number of features. LB and UB are the limits of the search domain. rand(1,D) stands for the random value with *D* values.

The next step is to generate the Boolean version of $X_{i}$ using the following formula:

(19) $$BX_{ij}=\left\{\begin{array}{cc} 1 & if\;X_{ij}>0.5\\ 0 & otherwise \end{array}\right.$$

We select only the features corresponding to ones in $BX_{i}$ and remove the other features. Then, we compute the fitness value of $X_{i}$ as:

(20) $$Fit_{i}=\left(1-\lambda \right)\times \left(\frac{|BX_{i}|}{D}\right)+\lambda \times \gamma_{i}$$

where $\gamma_{i}$ stands for the classification error based on KNN using the training sets, whereas, λ∈[0,1] is the weight used to control the balancing between $\gamma_{i}$ and the $\left(\frac{|BX_{i}|}{D}\right)$, which represents the ratio of relevant features.

Thereafter, we determined the best solution $X_{b}$ and used it to enhance the current solutions by combining GO and WOA. This was conducted using GO operators in the exploration phase, while the following integration schema was used during the exploitation phase.

(21) $$X_{i}=\left\{\begin{array}{cc} X_{WOA} & if\;Pr>r_{s}\\ X_{GO} & otherwise \end{array}\right.$$

where $X_{GO}$ refers to using the operators of GO that were used to update $X_{i}$ and $X_{WOA}$ is the operator of WOA defined in Equations (10)–(15). Pr is the probability of each $X_{i}$ and it is defined as:

(22) $$Pr=\frac{Fit_{i}}{\sum_{i=1}^{N}X_{i}}$$

where $Fit_{i}$ stands for the fitness value of $X_{i}$. In addition, the value of $r_{s}$ is updated using the following formula.

(23) $$r_{s}=min\left(Pr\right)+rand\times \left(max\left(Pr\right)-min\left(pr\right)\right)$$

where min and max are the minimum and maximum functions, respectively.

Then, the stop condition is checked and in case they are met, the update process is stopped. Otherwise, we repeat it again. After that, $X_{b}$ is used to remove irrelevant features from the testing set and evaluate this process using different performance criteria.

The time complexity of the developed MGO as FS method depends on some factors such as (1) the size of population *N*, (2) the dimension of features *D*, and (3) the number of iterations $t_{max}$. So, the complexity of MGO is formulated as:

(24) $$O\left(MGO\right)=N+t\times (N\times D+\left(O\left(WOA\right)+O\left(GO_{Reflection}\right)\right)\times D)$$

(25) $$O\left(MGO\right)=N\times D+t\times (N\times D+\left(K_{w}\times D+\left(N-K_{w}\right)\times D\right))$$

So,

(26) O(MGO)=N×D+t×(N×D)=O(N×D×t)

where $K_{w}$ refers to the number of solutions that will be updated using WOA.

## 4. Experimental Series and Results

The section uses a set of experimental series to assess the developed IoT security based on a modified version of the GO algorithm using WOA. These experimental series are implemented using a set of real-world IoT datasets.

### 4.1. Evaluation Measures

The effectiveness of the suggested technique and all comparing methodologies is evaluated using several indicators.

- Average accuracy ($AV_{Acc}$: This measure stands for the rate of a correct intrusion detected using the algorithm and it is represented as:
  (27) $$AV_{Acc}=\frac{1}{N_{r}}\sum_{k=1}^{N_{r}}Acc_{Best}^{k},\;$$
  $$Acc_{Best}=\frac{TP+TN}{TP+FN+FP+TN}$$
  in which $N_{r}=30$ indicates the iteration numbers.
- Average Recall $\left(AV_{recall}\right)$: is the percentage of intrusion predicted positively (it can be called true positive rate (TPR)). It can be computed as:
  (28) $$AV_{Recall}=\frac{1}{N_{r}}\sum_{k=1}^{N_{r}}Recall_{Best}^{k},\;Recall_{Best}=\frac{TP}{TP+FN}$$
- Average Precision $\left(AV_{Prec}\right)$: stands for the rate of TP samples of all positive cases with the formulation:
  (29) $$AV_{Prec}=\frac{1}{N_{r}}\sum_{k=1}^{N_{r}}Prec_{Best}^{k},Prec_{Best}=\frac{TP}{FP+TP}$$
- Average F1-measure $\left(AV_{F1}\right)$: can be computed as:
  (30) $$AV_{F1}=2\frac{Recall\times Precision}{Precision+Recall}$$
- Average G-mean $\left(AV_{GM}\right)$: can be computed as:
  (31) $$AV_{GM}=\sqrt{Recall\times Precision}$$

### 4.2. Experiments Setup

In our experiments, we trained the CNN model on each dataset record for 100 epochs with early stopping and Adam with a 0.005 learning rate to update the network parameters. A batch of size 2024 is used to iterate over the data samples. In addition, batch normalization and dropout with a 0.38 ratio were used as regularization techniques to prevent overfitting, increase generalization, and accelerate the training. The network hyper-parameters were selected based on several experiments with different setups where the best hyper-parameters were used in our experiments that maximize the detection accuracy. The CNN was developed using Pytorch framework (https://pytorch.org/, accessed on 15 January 2023) and the training was performed using Nvidia GTX 1080.

To test the performance of the developed MGO, we compared it to several optimizers, namely, the traditional WOA [21], the traditional GO, grey wolf optimizer (GWO) [23], Transient Search Optimization (TSO) [24], firefly algorithm (FFA) [25], and moth flame optimization (MFO) [26]. We set the parameters of each algorithm based on its original implementation, whereas the iterations number is set to 50, and the agent number is 20.

### 4.3. Experimental Datasets

**Figure 3:**
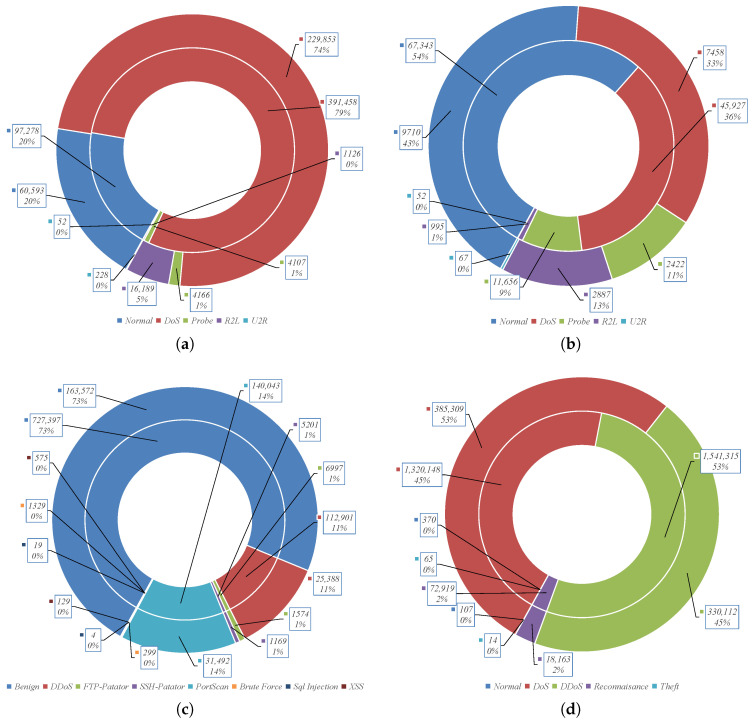
Datasets statistics where the Test set is the outer circle and the Train set is the inner circle. (**a**) KDDCup-99. (**b**) NSL-KDD. (**c**) CICIDS-2017. (**d**) Bot-IoT.

To validate the proposed framework, we used four well-known datasets for network intrusion detection, which are publicly available. The datasets used to train and test the proposed framework are KDDCup-99, NSL-KDD, BoT-IoT, and CICIDS-2017. Figure 3 shows the corresponding statistics of the datasets used in our experiments. The KDD (Knowledge Discovery and Data Mining) Cup 1999 dataset (KDDCup-99) was created in 1999 for the KDD cup competition organized by the Defence Advanced Research Project Agency (DARPA). The KDDCup-99 collects TCP/IP dump files containing network traffic records recorded for two months. The total number of records is around five million, with 41 features. We used the 10% version of the KDDCup-99 dataset, which contains less than one million records with four attack types, including user-to-root (U2R), probing, remote-to-user (R2L), and denial-of-service (DoS) besides normal traffic. The NSL-KDD dataset is a distilled version of the KDDCup-99 dataset with 41 features. The CICIDS-2017 [27] dataset provides more realistic network traffic records with 79 features collected by the CICFlowMeter tool focusing on SSH, HTTP, HTTPS, email, and FTP protocols. The dataset is presented in several CSV files, where we used four files to train and test the framework. The total used network records are around 600 thousand, with seven attack types and normal traffic. For the Bot-IoT dataset [28], we used the 5% version with 3.5 million records collected from various IoT devices.

### 4.4. Result and Discussions

This section discusses the comparison results between the developed MGO and other methods to improve the quality of IoT security. Table 1 shows the average over 25 independent runs for each algorithm using the performance measures.

In the multi-classification analysis, the MGO algorithm demonstrates superior efficiency compared to other algorithms during the learning phase on the datasets (i.e., KDD99, NSL-KDD, BIoT, and CIC2017). However, it falls behind the RSA regarding performance on the BIoT dataset. Furthermore, MGO excels in detecting attack types using testing samples on all four datasets compared to other methods.

In addition, the accuracy value of RSA is better than other methods, followed by MFO which allocates the third rank overall to the other methods during the training stage, whereas the Accuracy of RSA based on the testing set is the best after the developed MGO algorithm. Based on Precision, F1-Measure, and Recall, the FFA, GWO, and MFO is the best algorithm that allocates the second rank among the tested algorithms within the testing phase.

**Figure 4:**
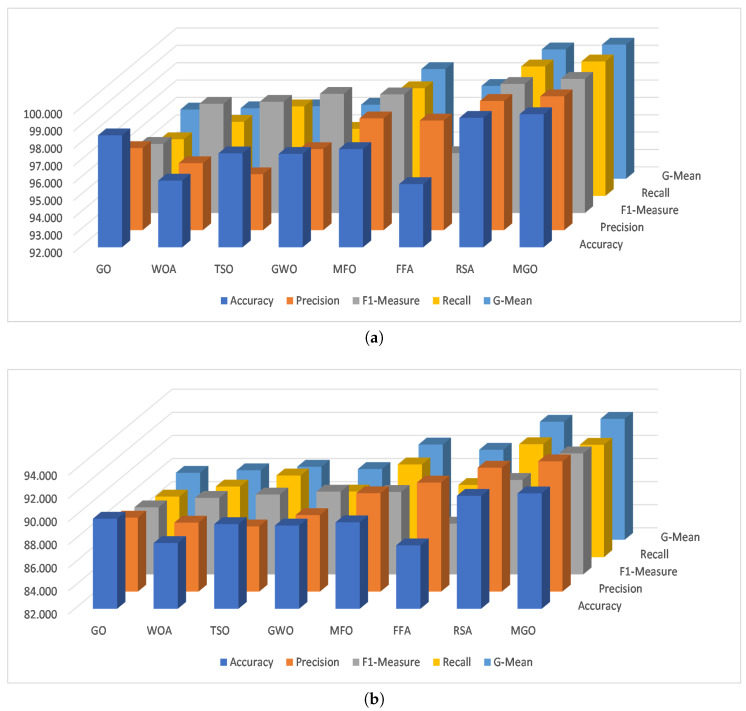
The average overall of the tested sets for multi-classification. (**a**) Train Set. (**b**) Testing set.

Figure 4 illustrates the average performance of each method across various datasets for various measures. The MGO method has the highest average performance for training and testing in multi-classification, followed by the Accuracy method which has better accuracy. The RSA method has better Recall in both training and testing, and GWO has a better F1-Measure in both training and testing. MFO and FFA have higher Precision in training and testing sets, respectively.

To further analyze the results, we applied the Friedman test to determine if there was a significant difference between the different methods. The Friedman test gave us the mean rank for each method, as seen in Table 2. From these mean ranks, we can see that MGO has the highest mean rank across all training and testing set performance measures, followed by RSA overall performance measures in case of using the training set. Meanwhile, the mean rank of the accuracy, precision, F1-measure, recall, and GM for the GO, FFA, MFO, TSO, and FFA, respectively, is the second rank after the developed MGO in the case of the testing set.

### 4.5. Comparison with Existing Methods

This section compares the results of the developed MGO with other techniques as given in Table 3. Most of those methods may use either one or two datasets. From these results, we can observe that in the case of KDD99, the accuracy of MGO is better than the method applied in [29]; however, the method presented in [30] has better performance than MGO. In the case of the BIoT, we can observe that the developed MGO performs better than other methods mentioned in this study, followed by the method introduced in Churcher et al. [31] ( KNN) which is superior to other methods. For the CICIDS2017 dataset, we noticed that MGO provided better results than the competitive methods.

From the previous results, it is clear that the developed method has a high potential to improve the prediction of attacks in IoT environments. However, the method has some limitations, such as being time-consuming due to the model learning process. These limitations can be addressed by using transfer learning techniques. In addition, the MGO still requires handling the imbalanced datasets in IoT and this can be handled by using the mechanism mentioned in [40].

## 5. Conclusions and Future work

Our study investigated the development of a two-phase framework to improve the detection accuracy over existing intrusion detection systems (IDS). In addition, the developed framework integrates a deep learning (DL) model and swarm intelligence (SI) technique to combine both techniques’ advantages and facilitate the deployment of the framework in the Internet of Things (IoT) system. We implemented a convolutional neural network architecture as a core feature extraction module to learn and extract new feature representation from the raw input data (network traffic records). In addition, we proposed a novel feature selection (FS) approach based on a modified variant of the Growth Optimizer (GO) algorithm to reduce the extracted feature representation space, speed up the inference, and improve the overall framework performance on IDS. The proposed FS method relies on applying the GWO to boost the search process of the traditional GO algorithm. Thus, the results show that the suggested method performed best compared to several optimization techniques using different evaluation indicators with several public IDS datasets. In future work, the developed MGO can be extended and experimented with in different applications such as healthcare, human activity recognition, fake news detection, and others.

## Figures and Tables

**Table 1:** The performance of MGO using the IoT security datasets. (Note: bold indicate best results).

		Train					Test				
		**Accuracy**	**Precision**	**F1**	**Recall**	**GM**	**Accuracy**	**Precision**	**F1**	**Recall**	**GM**
KDD99	GO	98.515	93.483	92.652	91.835	92.655	90.615	84.249	83.797	83.350	83.798
	WOA	92.275	92.414	97.304	93.126	92.769	84.375	82.501	87.351	85.225	83.852
	TSO	95.439	91.027	97.437	94.919	92.953	87.536	80.791	87.479	87.016	83.846
	GWO	95.513	94.062	98.482	92.383	93.219	87.618	84.131	88.533	84.488	84.310
	MFO	96.073	97.631	98.371	97.123	97.377	88.175	87.763	88.420	89.225	88.491
	FFA	91.988	97.328	91.538	93.368	95.328	84.318	91.609	84.285	85.698	88.604
	RSA	**99.910**	99.909	99.906	99.910	99.910	**92.040**	89.684	**89.985**	**92.040**	90.855
	MGO	**99.910**	**99.959**	**99.946**	**99.933**	**99.946**	**92.040**	**90.841**	90.941	91.040	**90.941**
NSL-KDD	GO	97.108	95.104	93.017	91.020	93.040	70.224	72.200	69.794	67.544	69.833
	WOA	91.947	92.080	96.968	92.797	92.438	67.951	71.131	68.907	68.801	69.956
	TSO	95.078	90.657	97.067	94.558	92.587	71.330	71.298	69.697	70.810	71.053
	GWO	95.182	93.724	98.143	92.052	92.884	71.066	72.151	69.948	67.936	70.012
	MFO	95.745	97.297	98.035	96.795	97.046	71.626	76.122	69.844	72.676	74.379
	FFA	91.660	96.991	91.201	93.040	94.995	67.437	75.873	62.944	68.817	72.259
	RSA	99.201	99.158	99.148	99.201	99.180	76.107	82.171	71.731	76.107	79.081
	MGO	**99.214**	**99.458**	**99.437**	**99.416**	**99.437**	**76.725**	**83.105**	**79.759**	**76.672**	**79.824**
BIoT	GO	99.068	99.107	99.076	99.045	99.076	99.141	98.100	98.371	98.644	98.372
	WOA	99.472	99.472	99.472	99.472	99.472	98.956	98.957	99.005	98.964	98.960
	TSO	99.460	99.459	99.459	99.460	99.460	98.986	98.941	99.005	98.981	98.961
	GWO	99.477	99.476	99.476	99.477	99.477	98.990	98.975	99.019	98.959	98.967
	MFO	99.480	99.480	99.480	99.480	99.480	98.998	99.013	99.020	99.009	99.011
	FFA	99.479	99.478	99.478	99.479	99.478	98.954	99.007	98.949	98.968	98.987
	RSA	98.829	98.829	98.829	98.829	98.829	99.020	99.098	99.070	99.038	99.068
	MGO	**99.629**	**99.529**	**99.629**	**99.729**	**99.629**	**99.220**	**99.188**	**99.218**	**99.248**	**99.218**
CIC2017	GO	99.130	99.239	99.204	99.170	99.204	99.170	99.020	99.215	99.410	99.215
	WOA	99.690	99.490	99.450	99.690	99.590	99.430	99.240	99.190	99.430	99.335
	TSO	99.680	99.750	99.680	99.710	99.730	99.420	99.480	99.420	99.450	99.465
	GWO	99.370	99.430	99.380	99.560	99.495	99.110	99.180	99.120	99.300	99.240
	MFO	99.360	99.370	99.480	99.430	99.400	99.100	99.120	99.220	99.170	99.145
	FFA	99.450	99.480	99.600	99.740	99.610	99.200	99.220	99.350	99.490	99.355
	RSA	99.911	99.910	99.889	99.911	99.910	99.911	99.907	99.888	99.911	99.909
	MGO	**99.941**	**99.920**	**99.926**	**99.931**	**99.926**	**99.941**	**99.947**	**99.942**	**99.936**	**99.942**

**Table 2:** Results of Friedman test.

		GO	WOA	TSO	GWO	MFO	FFA	RSA	MGO
Training	Accuracy	3.7500	3.5000	3.5000	4.0000	4.7500	3.0000	5.6250	7.8750
	Precision	2.5000	3.2500	2.7500	3.7500	5.2500	5.0000	5.5000	8.0000
	F1-Measure	1.7500	3.2500	4.2500	4.7500	5.2500	3.2500	5.5000	8.0000
	Recall	1.2500	3.5000	4.5000	3.0000	5.2500	5.0000	5.5000	8.0000
	GM	2.0000	2.7500	3.5000	3.7500	5.2500	5.2500	5.5000	8.0000
Testing	Accuracy	4.7500	3.0000	4.0000	3.5000	4.2500	1.7500	6.8750	7.8750
	Precision	2.5000	2.7500	2.7500	3.2500	4.7500	5.5000	6.7500	7.7500
	F1-Measure	2.2500	2.6250	4.1250	4.5000	5.0000	2.5000	7.0000	8.0000
	Recall	1.5000	3.2500	5.0000	2.0000	4.7500	4.5000	7.2500	7.7500
	GM	1.2500	2.7500	3.7500	3.5000	4.5000	5.2500	7.0000	8.0000

**Table 3:** Comparison with other methods.

Dataset	Work	Accuracy
KDD Cup 99	Wu [29]	85.24
	Farahnakian et al. [30]	96.53
	MGO	0.9204
NSL-KDD	Ma et al. [32] SCDNN	72.64
	Javaid et al. [33] STL	74.38
	Tang et al. [34] DNN	75.75
	Imamverdiyev et al. [35] Gaussian–Bernoulli RBM	73.23
	MGO	76.725
BIoT	[36] (BiLSTM)	98.91
	Alkadi et al. [36] (NB)	97.5
	Alkadi et al. [36] (SVM)	97.8
	Churcher et al. [31] (KNN)	99
	Churcher et al. [31] (SVM)	79
	Churcher et al. [31] (DT)	96
	Churcher et al. [31] (NB)	94
	Churcher et al. [31] (RF)	95
	Churcher et al. [31] (ANN)	97
	Churcher et al. [31] (LR)	74
	MGO	99.22
CICIDS2017	Vinayakumar [37]	94.61
	Laghrissi et al. [38]	85.64
	Alkahtani et al. [39]	80.91
	MGO	99.941

## Data Availability

All the datasets are public, as we described in the main text.
